# Decrease of dipeptidyl peptidase 4 activity is associated with weight loss after bariatric surgery

**DOI:** 10.1007/s11695-020-05200-0

**Published:** 2021-02-04

**Authors:** Carsten T. Herz, Johanna M. Brix, Bernhard Ludvik, Guntram Schernthaner, Gerit-Holger Schernthaner

**Affiliations:** 1 Division of Endocrinology and Metabolism, Department of Medicine III, Medical University of Vienna, Vienna, Austria; 2 Department of Medicine I, Klinik Landstraße, Vienna, Austria; 3 Division of Angiology, Department of Medicine II, Medical University of Vienna, Vienna, Austria; 4 Karl Landsteiner Institute for Obesity and Metabolic Disorders, Vienna, Austria

**Keywords:** dipeptidyl peptidase 4, morbid obesity, lipids, liver

## Abstract

**Purpose:**

Dipeptidyl peptidase 4 (DPP4) is expressed and secreted by adipocytes. DPP4 induces insulin resistance independently of its effect on glucagon-like peptide 1, thus it is conceivable that DPP4 directly contributes to metabolic dysfunction in patients with morbid obesity. The aim of this study was to investigate the impact of weight loss induced by bariatric surgery on DPP4 activity, and whether these changes are associated with improvements in markers of metabolic dysfunction and fatty liver disease.

**Materials and Methods:**

We included 68 non-diabetic patients who underwent bariatric surgery. Serum DPP4 activity was measured using a fluorogenic substrate before and after surgery.

**Results:**

Results: After a median follow-up period of 12 (IQR 11-17) months, median serum DPP4 activity decreased from 230 (IQR: 194-273) to 193 (164-252) pmol/min (p=0.012). The decrease in DPP4 activity was significantly correlated with decreases in BMI, improved cholesterol levels, reduced hepatic injury markers as well as improved post-prandial insulin sensitivity. After multivariable adjustment, ΔDPP4 activity remained significantly associated with Δcholesterol (beta=0.341, p=0.025), ΔLDL cholesterol (beta=0.350, p=0.019), Δgamma-glutamyltransferase (beta=0.323, p=0.040) and ΔMatsuda index (beta=-0.386, p=0.045).

**Conclusion:**

We demonstrated that weight loss induced by bariatric surgery results in decreased circulating DPP4 activity beyond the initial phase of weight loss. The associations between decreased DPP4 activity and improved cholesterol levels as well as hepatic injury markers point towards pleiotropic effects of DPP4 beyond glucose metabolism which warrant further investigation.

## Introduction

In the presence of an exceedingly positive energy balance, adipose tissue, especially the visceral depot, acts as a metabolic sink through expanding by hypertrophy resulting in an increasing whole body adiposity (1). It is hypothesized, that above a certain upper level of hypertrophy, adipose tissue hypoxia causes a local inflammatory reaction resulting in the secretion of cytokines, so-called adipokines, which is in turn aggravated by a subsequent infiltration of immune cells (2). These adipokines, such as tumor necrosis factor ɑ, plasminogen activator inhibitor 1, monocyte chemotactic protein 1, or interleukin 6, act systematically and have a negative influence on insulin sensitivity, beta cell function, coagulant/thrombotic state as well as endothelial function (3–5).

Dipeptidyl peptidase 4 (DPP4), a serine protease which degrades a range of incretins, growth factors, and cytokines, is secreted by lymphocytes, endothelial cells, smooth muscle cells, hepatocytes, as wells as adipocytes (6). Its expression is fivefold higher in visceral compared to subcutaneous fat, and individuals with obesity exhibit higher circulating levels compared to lean subjects (7). In metabolic research, DPP4 is mainly recognized as a negative regulator of the activity of the incretin glucagon-like peptide 1 (8). Beyond its indirect effects on glucose homeostasis, DPP4 has been shown to directly impair insulin signaling in adipocytes, skeletal muscle cells as well as in hepatocytes *in vitro* (7, 9). Pharmacological DPP4 inhibitors are now frequently prescribed for glycemic control in patients with type 2 diabetes mellitus (10).

Bariatric surgery is an effective treatment for morbid obesity leading to the improvement of its cardiometabolic sequelae such as type 2 diabetes mellitus, arterial hypertension, and hyperlipidemia (11, 12). Changes in a wide range of hormones have been proposed to mediate the metabolic benefits seen with bariatric surgery such as changes in postprandial incretin response or altered bile acid metabolism (13). Studies investigating the potential involvement of plasma DPP4 activity in bariatric surgery-associated metabolic improvements are, however, scarce. Thus, the aim of this study was to investigate the impact of bariatric surgery-induced weight loss on circulating DPP4 activity and whether changes in DPP4 activity are associated with the observed metabolic improvements.

## Methods

The patients were recruited in an ongoing observational study at the obesity outpatient clinic of the Department of Medicine I of Klinik Landstraße, formerly known as Krankenanstalt Rudolfstiftung, in Vienna, Austria. We recruited all consenting consecutive patients who were evaluated for bariatric surgery. Medical history, blood draws and anthropometric measures were taken before and at each routine follow-up visit after surgery. The patients either underwent laparoscopic Rouy-en-Y gastric bypass surgery or sleeve gastrectomy. The study was approved by the institutional ethics committee and complies with the Declaration of Helsinki including current revisions and the Good Clinical Practice guidelines. The procedures performed were in accordance with institutional guidelines and all subjects gave written informed consent before the study. For this sub-study, we selected 68 patients without previously known type 2 diabetes for whom pre- and post-operative serum samples were available. We excluded patients with previously known and already treated diabetes to exclude confounding due to changes in antidiabetic medications after surgery. Normal glucose metabolism, pre-diabetes and overt type 2 diabetes mellitus were diagnosed according to current guidelines (14). In a subset of 34 participants, results of an oral glucose tolerance tests (OGTT), performed after the ingestion of 75g glucose diluted in 300ml water with insulin and glucose measurements at 0, 60, 120 minutes, were available both before as well as after surgery.

All patients with severe renal or liver disease or patients on a systemic corticosteroid drug therapy were excluded from this study. In addition, all patients with acute psychiatric illnesses were excluded.

### Laboratory measurements

Peripheral venous blood samples were collected from all patients after an overnight fast for determination of basic laboratory characteristics. Routine laboratory measurements were performed at the institution's central laboratory. Homeostasis model assessment insulin resistance (HOMA-IR) index and Matsuda insulin sensitivity index were calculated using published formulae (15, 16). The Matsuda insulin sensitivity index is calculated from fasting as well as post-load glucose and insulin levels during an OGTT and has been shown to be highly correlated with whole-body glucose disposal during a hyperinsulinemia euglycemic clamp, the gold standard for the assessment of whole body insulin sensitivity (16). HOMA-IR, on the other hand, which is derived from fasting glucose and insulin measurements is thought to primarily reflect hepatic insulin resistance (15). Serum samples for the determination of DPP4 activity were centrifuged between 30 - 60 minutes after the blood draw and stored at -80°C. Serum DPP4 activity was assayed by a commercially available kit measuring the fluorescence of the cleaved fluorogenic DPP4 substrate H-Gly-Pro-AMC using a microplate reader (Enzo Life Sciences, Inc., Lausen, Switzerland).

#### Statistics

The data are presented as count, median (25^th^ - 75^th^ percentile), or mean ± standard deviation, as appropriate. Accordingly, within-groups differences were tested using Student’s paired t-test or Wilcoxon signed-rank test. To investigate associations between two continuous variables, Spearman’s rank correlation coefficient was used. To investigate longitudinal associations, change scores (∆) were calculated by subtracting the baseline value from the follow-up value of each respective variable.

We used multiple linear regression to adjusted associations between change scores for their respective baseline values and other variables of interest. Effect size is given as standardized regression coefficients (beta). All analyses were performed using SPSS 25 (IBM Corp., Armonk, NY). Two-sided p-values ≤0.05 were deemed statistically significant.

## Results

This study included 68 individuals with morbid obesity of which 53 (78%) were female with a mean age of 42 ± 12 years and a mean BMI of 46.9 ± 7.7 kg/m^2^. The median baseline HbA_1C_ value was 5.8 (IQR: 5.5 - 6.3) % indicating an impaired glucose metabolism in more than half of the participants: 31 (45.6%) fulfilled the diagnostic criteria for prediabetes and 14 (20.6%) for diabetes. The remaining baseline characteristics are depicted in table 1. Among the baseline clinical variables depicted in table 1, baseline DPP4 activity correlated with the hepatic markers alanine aminotransferase (ALAT) (rho=0.274, p=0.026) and gamma-glutamyltransferase (GGT) (rho=0.280, p=0.002) while there were no significant correlations with BMI (rho=0.082, p=0.508) or the marker of insulin resistance HOMA-IR (rho=0.172, p=0.175).

**Table 1.** Patients’ characteristics before and after bariatric surgery

	baseline	post surgery	p
BMI (kg/m^2^)	46.9 ± 7.7	30.3 ± 9.3	< 0.001
Glucose (mg/dL)	100 ± 19	91 ± 20	0.001
Insulin (μU/mL)	25 (16 - 35)	9 (6 - 15)	< 0.001
HOMA-IR	6.2 (3.8 - 8.1)	2 (1.2 - 3.3)	< 0.001
Matsuda index	1.56 (1.02 - 2.78)	5.22 (3.41 - 7.78)	< 0.001
HbA_1C_ (%)	5.8 (5.5 - 6.3)	5.3 (5 - 5.5)	< 0.001
Triglycerides (mg/dL)	136 (96 - 178)	81 (65 - 119)	< 0.001
Cholesterol (mg/dL)	200 ± 33	185 ± 32	0.001
LDL cholesterol (mg/dL)	123 ± 30	110 ± 29	0.001
HDL cholesterol (mg/dL)	47 ± 11	56 ± 14	< 0.001
GGT (U/L)	28 (18 - 36)	15 (9 - 20)	< 0.001
ALAT (U/L)	29 (22 - 41)	19 (16 - 27)	< 0.001
ASAT (U/L)	22 (19 - 29)	20 (16 - 23)	0.020
C-reactive protein (mg/L)	6.9 (4 - 1.1)	2 (1 - 4.6)	< 0.001

ASAT: aspartate aminotransferase; ALAT: alanine aminotransferase; BMI: body mass index; GGT: gamma-glutamyltransferase; HbA_1C_: glycated hemoglobin; HOMA-IR: homeostasis model assessment of insulin resistance index; HDL: high-density lipoprotein; LDL: low-density lipoprotein. Data are depicted as counts, medians (25^th^ - 75^th^ percentile), or mean ± standard deviation. Between group comparisons were performed Student’s paired sample t-test or Wilcoxon signed-rank test, as appropriate.

**Fig. 1.**
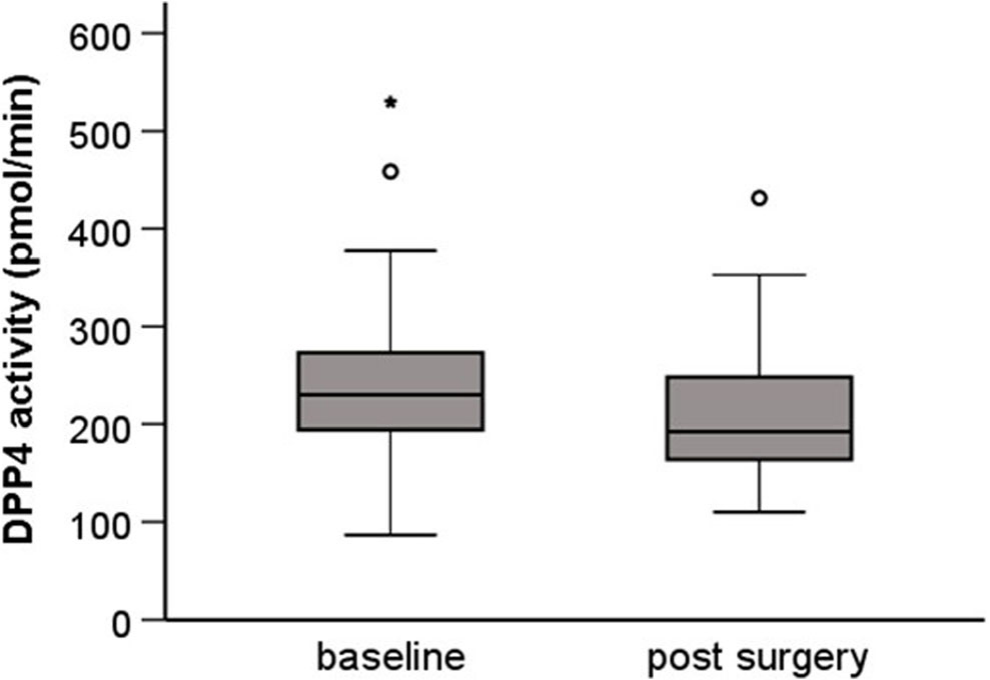
Boxplot for the comparison between pre- and post-surgical values of serum DPP4 activity. * p≤0.005; °outlier

Sleeve gastrectomy and laparoscopic Roux-en-Y gastric bypass surgery were performed in 58 (85.3%) and 10 (14.7%) patients, respectively. After a median follow-up of 12 (IQR 11 – 17) months, mean weight loss was 43 (95%CI: 40 - 47) kg of body weight corresponding to a change in BMI from 46.9 ± 7.7 to 30.3 ± 9.3 kg/m^2^. As expected, this was accompanied by relevant improvements in cardio-metabolic risk markers, and liver parameters (table 1). At the same time, DPP4 activity declined from 230 (IQR: 194 - 273) to 193 (164 - 252) pmol/min (p = 0.012, Figure 1). The decrease in DPP4 activity did not significantly differ between normoglycemic, prediabetic or diabetic patients after adjusting for baseline DPP4 activity (p=0.125). When investigating the associations between postoperative changes, the decrease in DPP4 activity correlated significantly with the changes in BMI (rho=0.286, p=0.023), total cholesterol (rho=0.410, p=0.001), LDL cholesterol (rho=0.383, p=0.003), ALAT (rho=0.380, p=0.003), ASAT (rho=0.295, p=0.034), GGT (rho=0.276, p=0.034) but not with the change in HOMA-IR (rho=0.094, p=0.503). In the subset of participants who underwent an oral glucose tolerance test, ΔDPP4 activity correlated with ΔMatsuda index (rho=-0.475, p=0.005).

We used multivariable linear regression to investigate whether the associations between ΔDPP4 activity and the beneficial changes in metabolic markers were independent of the loss of body mass. After adjusting for baseline DPP4 activity, baseline BMI, change in BMI, and the baseline values of the respective independent variable, ΔDPP4 activity was associated with Δtotal cholesterol (beta=0.341, p=0.025), ΔLDL cholesterol (beta=0.350, p=0.019), ΔGGT (beta=0.323, p=0.040), and ΔMatsuda index (beta=-0.386, p=0.045) but not with ΔALAT (beta=0.118, p=0.357), ΔASAT (beta=-0.028, p=0.827).

## Discussion

In the present study, we found that plasma DPP4 activity is significantly reduced after weight loss induced by bariatric surgery. As bariatric surgery is associated with loss of a large portion of excess body fat (17), this finding is in accordance with the notion that a substantial part of circulating DPP4 levels are derived from adipose tissue. Previous data indicate that decreased DPP4 levels after weight loss not only reflect the reduction in adipose tissue mass *per se* but also an ameliorated adipocyte hypertrophy (7). Other studies investigating the impact of gastric bypass surgery on circulating DPP4 activity rather than serum levels reported divergent results. In small cohorts, *Alam et al.* reported a decrease in DPP4 activity by 11.6 % one month after gastric bypass (n=16) while *Sarkar et al* found no differences in DPP4 activity 4-6 weeks after gastric bypass surgery (n=20) (18, 19). Studies investigating early, sometimes transient, metabolic changes are important to assess mechanisms independent of weight loss contributing to diabetes remission. The longer follow-up period of our study reflected the persistent changes in metabolism after weight stability is usually achieved. We can however draw no conclusions about the weight-loss independent effects on DPP4 activity. At the same time, our findings are not confounded by transient metabolic alterations observed within the first months after surgery owing to a catabolic state with an excessively negative energy balance characterized by a metabolism mostly relying on lipid oxidation and ketogenesis (20).

Interestingly, the decrease in DPP4 activity was not associated with improved fasting insulin resistance, as indicated by HOMA-IR, but rather with increasing post-prandial insulin sensitivity, as estimated by the Matsuda index. However, our study lacks insulin resistance measures derived from the gold standard, the hyperinsulinemic euglycemic clamp, and thus replication using this technique is needed. Still, this finding hints towards a potential contribution of weight loss-induced decrease in DPP4 to improved incretin response. Lower circulating DPP4 activity following bariatric surgery could, besides hypertrophy of intestinal L-cells, be an additional explanatory factor for the large increase in post-prandial GLP-1 levels after bariatric surgery (21, 22). Augmented incretin response is believed to be an important contributor to the amelioration of glycemic control achieved by bariatric surgery (23). However, the association between changes in DPP4 activity and GLP-1 levels could not be addressed in the present study due to the lack of post-prandial serum samples. We further found that the decreasing DPP4 activity after surgery is associated with an improved atherogenic lipid profile, especially with decreasing atherogenic LDL cholesterol levels, independently of weight loss. It is a well observed fact that bariatric procedures significantly reduce levels of cholesterol and LDL cholesterol (24). Among the hypothesized mechanisms contributing to reduced cholesterol levels after bariatric surgery are a decreased intestinal cholesterol absorption, an increased trans-intestinal cholesterol excretion as well as an increased flux of cholesterol into bile acid synthesis due to augmented fecal excretion of bile acids (25, 26). Interestingly, there are studies suggesting a direct role of DPP4 in the regulation of cholesterol levels. A study in mice showed that the DPP4 inhibitor sitagliptin increases fecal cholesterol loss by reducing intestinal cholesterol absorption and increasing macrophage-to-feces reverse cholesterol transport (27). Two other mouse studies with sitagliptin and anagliptin, respectively, provide divergent conclusions as to whether this effect depends on increased GLP-1 signaling (28, 29). A clinical trial in patients with type 2 diabetes, however, found no effect of anagliptin on cholesterol absorption markers. After one month of treatment, anagliptin significantly decreased the cholesterol synthesis marker lathosterol while no changes in the absorption markers campesterol, sitosterol or cholestanol could be observed (30). Accordingly, our study provides evidence that decreased DPP4 activity after weight loss surgery might contribute to the improvement in dyslipidemia.

Another finding in our study was an association of the decline of the DPP4 activity

with the early liver marker GGT. Bariatric surgery has already been proven to lead to a histological resolution of non-alcoholic steatohepatitis (NASH) (31). NASH is a common comorbidity observed in morbidly obese patients and the consequence of excessive hepatic triglyceride accumulation secondary to visceral fat expansion and insulin resistance (32). Few clinical studies reported elevated circulating DPP4 activity in patients with NAFLD and NASH (9, 33–36). Circulating DPP4 activity correlated significantly with caspase-cleaved keratin-18, a hepatic apoptosis marker, with liver stiffness, as assessed by transient elastography, and the histological fibrosis stage (35). Additionally, DPP4 expression in hepatocytes has been shown to be correlated with histopathological grading of NASH (33). Interestingly, treatment with the DPP4 inhibitor des-fluoro-sitagliptin resulted in an amelioration of diet-induced visceral obesity and hepatic steatosis. On a molecular level, the authors described a reduced expression of the master transcriptional regulator of lipid synthesis sterol regulatory element–binding protein-1c as well as a reduced expression of the lipogenic enzymes stearoyl-CoA desaturase-1 and fatty acid synthase (37). Current literature further provides evidence for a causative rather than a purely consequential role of DPP4 in NAFLD/ NASH. Hepatic overexpression of DPP4 in mice on a high-fat diet led to hepatic insulin resistance accompanied by hepatic steatosis and liver damage as well as increased body weight, fat mass, adipose tissue inflammation, and hypercholesterolemia. *In vitro,* treatment of human hepatocyte lines with DPP4 in the physiological range resulted in an impaired insulin sensitivity (9). Taken together, these data point towards a reciprocal relationship between DPP4 and metabolic hepatic disease. A contribution of increased GLP-1 signaling to the observed effects cannot be excluded as studies have shown that direct treatment of hepatocytes with GLP-1 analogues suppresses lipogenesis and induces lipolysis (38). Taken together, reduced DPP4 activity after bariatric surgery could both be a marker of as well as a contributor to improvement in NAFLD at the same time.

The study has, however, some limitations. Due to the observational design, we cannot distinguish an association from a causal role. In addition, the measurement of GLP-1 levels, especially after a mixed-meal challenge, could have provided insights whether the observed findings were independent of GLP-1 signaling.

A strength of this study is the sample size, which is substantially larger than previously reported studies. Due to the exclusion of individuals with previously known and treated diabetes, we can rule out the effects of antidiabetic medications on DPP4 activity. Compared to previous studies, we chose a longer follow-up period after which patients usually are weight stable and thus lets us draw conclusions about the long-term metabolic effects of the intervention.

To summarize, we described a significant reduction in DPP4 activity following surgically induced weight loss which was associated both with an ameliorated dyslipidemia as was as an improvement in the liver marker GGT as an indicator for improved NAFLD/ NASH. The independence of the changes in weight and the lack of association with insulin resistance point towards direct involvements of DPP4 independent of the well-described effects on glucose metabolism.

## References

[1] Vishvanath L, Gupta RK (2019). Contribution of adipogenesis to healthy adipose tissue expansion in obesity. J Clin Invest..

[2] Trayhurn P (2013). Hypoxia and adipose tissue function and dysfunction in obesity. Physiol Rev..

[3] Liberale L, Bonaventura A, Vecchiè A, Casula M, Dallegri F, Montecucco F (2017). The Role of Adipocytokines in Coronary Atherosclerosis. Curr Atheroscler Rep..

[4] Satish M, Saxena SK, Adipokine Dysregulation ADK (2019). Insulin Resistance with Atherosclerotic Vascular Disease: Metabolic Syndrome or Independent Sequelae?. J Cardiovasc Transl Res..

[5] Kumari R, Kumar S, Kant R (2019). An update on metabolic syndrome: Metabolic risk markers and adipokines in the development of metabolic syndrome. Diabetes Metab Syndr..

[6] Nargis T, Chakrabarti P (2018). Significance of circulatory DPP4 activity in metabolic diseases. IUBMB Life..

[7] Lamers D, Famulla S, Wronkowitz N, Hartwig S, Lehr S, Ouwens DM (2011). Dipeptidyl peptidase 4 is a novel adipokine potentially linking obesity to the metabolic syndrome. Diabetes..

[8] Mentlein R, Gallwitz B, Schmidt WE (1993). Dipeptidyl-peptidase IV hydrolyses gastric inhibitory polypeptide, glucagon-like peptide-1(7-36)amide, peptide histidine methionine and is responsible for their degradation in human serum. Eur J Biochem..

[9] Baumeier C, Schlüter L, Saussenthaler S, Laeger T, Rödiger M, Alaze SA (2017). Elevated hepatic DPP4 activity promotes insulin resistance and non-alcoholic fatty liver disease. Mol Metab..

[10] Deacon CF (2020). Dipeptidyl peptidase 4 inhibitors in the treatment of type 2 diabetes mellitus. Nat Rev Endocrinol..

[11] Madadi F, Jawad R, Mousati I, Plaeke P, Hubens G (2019). Remission of Type 2 Diabetes and Sleeve Gastrectomy in Morbid Obesity: a Comparative Systematic Review and Meta-analysis. Obes Surg..

[12] Han Y, Jia Y, Wang H, Cao L, Zhao Y (2020). Comparative analysis of weight loss and resolution of comorbidities between laparoscopic sleeve gastrectomy and Roux-en-Y gastric bypass: A systematic review and meta-analysis based on 18 studies. Int J Surg..

[13] Docherty NG, Le Roux CW (2015). Physiological adaptations following Roux-en-Y gastric bypass and the identification of targets for bariatric mimetic pharmacotherapy. Curr Opin Pharmacol..

[14] American Diabetes Association. 2. Classification and Diagnosis of Diabetes (2020). Standards of Medical Care in Diabetes-2020. Diabetes Care..

[15] Matthews DR, Hosker JP, Rudenski AS, Naylor BA, Treacher DF, Turner RC (1985). Homeostasis model assessment: insulin resistance and beta-cell function from fasting plasma glucose and insulin concentrations in man. Diabetologia..

[16] Matsuda M, DeFronzo RA (1999). Insulin sensitivity indices obtained from oral glucose tolerance testing: comparison with the euglycemic insulin clamp. Diabetes Care..

[17] Merlotti C, Ceriani V, Morabito A, Pontiroli AE (2017). Subcutaneous fat loss is greater than visceral fat loss with diet and exercise, weight-loss promoting drugs and bariatric surgery: a critical review and meta-analysis. Int J Obes.

[18] Alam ML, Van der Schueren BJ, Ahren B, Wang GC, Swerdlow NJ, Arias S (2011). Gastric bypass surgery, but not caloric restriction, decreases dipeptidyl peptidase-4 activity in obese patients with type 2 diabetes. Diabetes Obes Metab..

[19] Sarkar J, Nargis T, Tantia O, Ghosh S, Chakrabarti P (2019). Increased Plasma Dipeptidyl Peptidase-4 (DPP4) Activity Is an Obesity-Independent Parameter for Glycemic Deregulation in Type 2 Diabetes Patients. Front Endocrinol.

[20] Müller MJ, Enderle J, Bosy-Westphal A (2016). Changes in Energy Expenditure with Weight Gain and Weight Loss in Humans. Curr Obes Rep..

[21] Jirapinyo P, Jin DX, Qazi T, Mishra N, Thompson CCA (2018). Meta-Analysis of GLP-1 After Roux-En-Y Gastric Bypass: Impact of Surgical Technique and Measurement Strategy. Obes Surg..

[22] Hansen CF, Bueter M, Theis N, Lutz T, Paulsen S, Dalbøge LS (2013). Hypertrophy dependent doubling of L-cells in Roux-en-Y gastric bypass operated rats. PLoS One..

[23] Russel SM, Valle V, Spagni G, Hamilton S, Patel T, Abdukadyrov N (2020). Physiologic Mechanisms of Type II Diabetes Mellitus Remission Following Bariatric Surgery: a Meta-analysis and Clinical Implications. J Gastrointest Surg..

[24] Qi L, Guo Y, Liu C-Q, Huang Z-P, Sheng Y, Zou D-J (2017). Effects of bariatric surgery on glycemic and lipid metabolism, surgical complication and quality of life in adolescents with obesity: a systematic review and meta-analysis. Surg Obes Relat Dis..

[25] Blanchard C, Moreau F, Ayer A, Toque L, Garçon D, Arnaud L (2018). Roux-en-Y gastric bypass reduces plasma cholesterol in diet-induced obese mice by affecting trans-intestinal cholesterol excretion and intestinal cholesterol absorption. Int J Obes.

[26] So SSY, Yeung CHC, Schooling CM, El-Nezami H (2020). Targeting bile acid metabolism in obesity reduction: A systematic review and meta-analysis. Obes Rev..

[27] Briand F, Thieblemont Q, Burcelin R, Sulpice T (2012). Sitagliptin promotes macrophage-to-faeces reverse cholesterol transport through reduced intestinal cholesterol absorption in obese insulin resistant CETP-apoB100 transgenic mice. Diabetes Obes Metab..

[28] Hsieh J, Longuet C, Baker CL, Qin B, Federico LM, Drucker DJ (2010). The glucagon-like peptide 1 receptor is essential for postprandial lipoprotein synthesis and secretion in hamsters and mice. Diabetologia..

[29] Goto M, Furuta S, Yamashita S, Hashimoto H, Yano W, Inoue N (2018). Dipeptidyl peptidase 4 inhibitor anagliptin ameliorates hypercholesterolemia in hypercholesterolemic mice through inhibition of intestinal cholesterol transport. J Diabetes Investig..

[30] Aoki K, Ijima T, Kamiyama H, Kamiko K, Terauchi Y (2015). Anagliptin decreases serum lathosterol level in patients with type 2 diabetes: a pilot study. Expert Opin Pharmacother..

[31] Lassailly G, Caiazzo R, Buob D, Pigeyre M, Verkindt H, Labreuche J, et al. Bariatric Surgery Reduces Features of Nonalcoholic Steatohepatitis in Morbidly Obese Patients. Gastroenterology. 2015 Aug;149(2):379–88; quiz e15-6. 10.1053/j.gastro.2015.04.014 25917783

[32] Stefan N, Häring H-U, Cusi K (2019). Non-alcoholic fatty liver disease: causes, diagnosis, cardiometabolic consequences. and treatment strategies. Lancet Diabetes Endocrinol..

[33] Balaban YH, Korkusuz P, Simsek H, Gokcan H, Gedikoglu G, Pinar A (2007). Dipeptidyl peptidase IV (DDP IV) in NASH patients. Ann Hepatol..

[34] Miyazaki M, Kato M, Tanaka K, Tanaka M, Kohjima M, Nakamura K (2012). Increased hepatic expression of dipeptidyl peptidase-4 in non-alcoholic fatty liver disease and its association with insulin resistance and glucose metabolism. Mol Med Rep..

[35] Williams KH, Vieira De Ribeiro AJ, Prakoso E, Veillard A-S, Shackel NA, Brooks B (2015). Circulating dipeptidyl peptidase-4 activity correlates with measures of hepatocyte apoptosis and fibrosis in non-alcoholic fatty liver disease in type 2 diabetes mellitus and obesity: A dual cohort cross-sectional study. J Diabetes..

[36] Zheng T, Chen B, Yang L, Hu X, Zhang X, Liu H (2017). Association of plasma dipeptidyl peptidase-4 activity with non-alcoholic fatty liver disease in nondiabetic Chinese population. Metabolism..

[37] Shirakawa J, Fujii H, Ohnuma K, Sato K, Ito Y, Kaji M (2011). Diet-induced adipose tissue inflammation and liver steatosis are prevented by DPP-4 inhibition in diabetic mice. Diabetes..

[38] Ben-Shlomo S, Zvibel I, Shnell M, Shlomai A, Chepurko E, Halpern Z (2011). Glucagon-like peptide-1 reduces hepatic lipogenesis via activation of AMP-activated protein kinase. J Hepatol..

